# Urban tree composition is associated with breeding success of a passerine bird, but effects vary within and between years

**DOI:** 10.1007/s00442-023-05319-8

**Published:** 2023-01-21

**Authors:** Johan Kjellberg Jensen, Johan Ekroos, Hannah Watson, Pablo Salmón, Peter Olsson, Caroline Isaksson

**Affiliations:** 1 Department of Biology, Lund University, Lund, Sweden; 2 Centre for Environmental and Climate Science (CEC), Lund University, Lund, Sweden; 3 Department of Agricultural Sciences, University of Helsinki, Helsinki, Finland; 4 Institute of Biodiversity, Animal Health and Comparative Medicine, University of Glasgow, Glasgow, UK

**Keywords:** Food limitation, Non-native trees, Parus major, Territory, Urbanization

## Abstract

Birds breeding in urban environments have lower reproductive output compared to rural conspecifics, most likely because of food limitation. However, which characteristics of urban environments may cause this deficiency is not clear. Here, we investigated how tree composition within urban territories of passerine birds is associated with breeding probability and reproductive success. We used 7 years of data of breeding occupancy for blue and great tits (*Cyanistes caeruleus; Parus major*) and several reproductive traits for great tits, from 400 urban nest boxes located in 5 parks within the city of Malmö, Sweden. We found that tits, overall, were less likely to breed in territories dominated by either non-native trees or beech trees. Great tit chicks reared in territories dominated by non-native trees weighed significantly less, compared to territories with fewer non-native trees. An earlier onset of breeding correlated with increased chick weight in great tits. Increasing number of common oak trees (*Quercus robur*) was associated with delayed onset of breeding in great tits. Notably, as offspring survival probability generally increased by breeding earlier, in particular in oak-dominated territories, our results suggest that delayed onset of breeding induced by oak trees may be maladaptive and indicate a mismatch to this food source. Our results demonstrate that tree composition may have important consequences on breeding success of urban birds, but some of these effects are not consistent between years, highlighting the need to account for temporal effects to understand determinants of breeding success and inform optimal management in urban green spaces.

**Supplementary Information:**

The online version contains supplementary material available at 10.1007/s00442-023-05319-8.

## Introduction

Urbanization rapidly alters local environments through a wide array of factors, resulting in novel habitats that may be suboptimal to breed in for many organisms (Grimm et al. 2008; Aronson et al. 2014). As an example, passerine birds breeding in urban environments generally produce fewer nestlings and nestlings of lower body mass compared to rural populations (Chamberlain et al. 2009). The driving factor of reduced reproductive output for passerine birds in urban habitats has long been hypothesized to be food limitation (Chamberlain et al. 2009), yet it is unclear what urban factors cause this limitation. Recent studies have confirmed that the diet of urban passerines is distinctly different compared with non-urban passerines (Pollock et al. 2017; Jarrett et al. 2020). In addition, great tits (*Parus major*) supplemented with nutritionally enriched mealworms have been shown to mitigate the negative urban effects on nestling body size and survival (Seress et al. 2020). While other factors in urban environments, including artificial light at night (Dominoni et al. 2020) and pollution (Eeva et al. 2009), affect avian reproduction, the magnitude of the food supplementation effect shown by Seress et al. (2020) suggests that food availability and/or quality is a major limiting factor for the reproductive success of urban insectivorous passerines. A lower availability of preferred prey items (i.e., lepidopteran caterpillars; see Naef-Daenzer et al. 2000) in urban environments compared to non-urban ones corroborates the food limitation hypothesis further (Pollock et al. 2017; Seress et al. 2018; Baldan and Ouyang 2020). Notably, the nestling diet of tit species has been observed to contain significantly fewer caterpillars in urban birds compared to rural birds (Pollock et al. 2017; Jarrett et al. 2020). In addition, urban caterpillars have also been found to have decreased levels of nutritional compounds such as carotenoids, suggesting that they are a lower quality food source (Isaksson and Andersson 2007).

However, it is still largely unknown which aspects of the urban habitat drive the variation in breeding success through possible food limitation for insectivorous birds. While some studies have looked at the effects of urbanization gradients and foraging behavior on avian reproduction (e.g., Caizergues et al. 2021; Jarrett et al. 2020), a high-resolution analysis of the biotic components in the immediate surroundings of breeding urban birds, here referred to as territory, and its effects on avian reproduction across years is still lacking (but see Narango et al. 2018). Analyzing the effects of tree composition on breeding success across multiple years could render important answers, as the availability of caterpillars and other invertebrates are often closely linked to the availability of host plants, in addition to being annually variable (van Asch and Visser 2007; Mutshinda et al. 2011). Furthermore, vegetation itself is one of the most important aspects for maintaining biodiversity in urban environments (Beninde et al. 2015). Whereas vegetation is arguably the major biotic component of the urban ecosystem which is managed to an impactful degree (Faeth et al. 2011), other factors, such as esthetics and presumed ease of maintenance, besides ecosystem health, are often prioritized in management decisions concerning urban green spaces (Avolio et al. 2018).

In particular, the planting of non-native vegetation within cities is widespread: currently, over a quarter of urban plant species are non-native (Aronson et al. 2014; van Kleunen et al. 2015). Non-native plants are known to host fewer invertebrates as a consequence of lacking a co-evolutionary history with the local ecosystem (Brändle et al. 2008; Padovani et al. 2020; Tallamy et al. 2021; Jensen et al. 2022), which in turn has been linked to negative consequences for birds (Burghardt et al. 2009; Narango et al. 2017, 2018). Specifically, suburban areas with a higher share of native plants have been linked to higher caterpillar abundance and diversity of bird species (Burghardt et al. 2009), while areas dominated by non-native vegetation are associated with lower breeding success for local birds, as they shift their diet to less preferred prey (Narango et al. 2018).

Urban environments affect animal and plant phenology by changes in ambient temperatures through the urban heat island effect (Dallimer et al. 2016; Li et al. 2019). An early onset of breeding tends to have a positive effect of reproductive success for birds in general (Villemereuil et al. 2020). Passerines relying on caterpillars as food source, such as the great tit, adjust their onset of breeding in order to match caterpillar phenology and thereby food availability (Lack 1950; van Noordwijk et al. 1995; Visser et al. 2006). Lepidopteran caterpillars are in turn closely tied to their host plants’ phenology. A slight mismatch in caterpillar emergence can have detrimental effects at the population level for lepidopterans, because leaves quickly decrease in water and nitrogen content, while defense compounds increase (van Asch and Visser 2007). In urban areas, non-native tree species differ from natives in phenology; while urban native trees show advanced budburst in relation to their rural counterparts in the Northern hemisphere, urban non-native trees were found to have a delayed phenology compared to native trees within the city (Jensen et al. 2022, Appendix S1). Combined with artificial light at night, ambient temperature has been found to modulate egg-laying phenology of great tits (Dominoni et al. 2020). Therefore, effects of tree composition in urban territories may have contrasting effects on the breeding success of birds depending on ambient temperature and the onset of breeding.

Here, we investigate local tree composition, which may affect the reproductive success of urban great tits, potentially through food limitation, across multiple years at a high-resolution spatial scale. We mapped tree composition around 400 nest boxes located throughout 5 city parks in the city of Malmö, Sweden, resulting in unique territory profiles for each nest box. Using 7 years of breeding data, we analyzed how tree composition in the local urban territory affected the likelihood of blue tit (*Cyanistes caeruleus*) and great tit breeding attempts and the breeding phenology, breeding success and nestling weight of great tits.

In line with previous studies, we hypothesized that the number of common oak trees (*Quercus robur*) would positively affect the probability of breeding attempts and success, and nestling weight in addition to modulating the breeding phenology (Naef-Daenzer et al. 2000; Visser et al. 2006; Narango et al. 2020). Furthermore, we expected increasing tree diversity to have a positive effect on breeding success and nestling weight, as we expected that it would increase the number of potential food sources within the territory, in addition to spreading out the food availability over a longer period. Since non-native trees host a lower abundance of invertebrates and caterpillars compared to native trees in our study system (Jensen et al. 2022), we expected the number of non-native tree individuals in the territory to have a negative effect on breeding success and nestling weight (Narango et al. 2018). Finally, we expected to find annual variation in the magnitude and direction of effects, as yearly differences in temperature and budburst may result in different food sources being available, in addition to territories varying in their local peak of food abundance.

## Methods

### Monitoring of bird populations

**Fig. 1:**
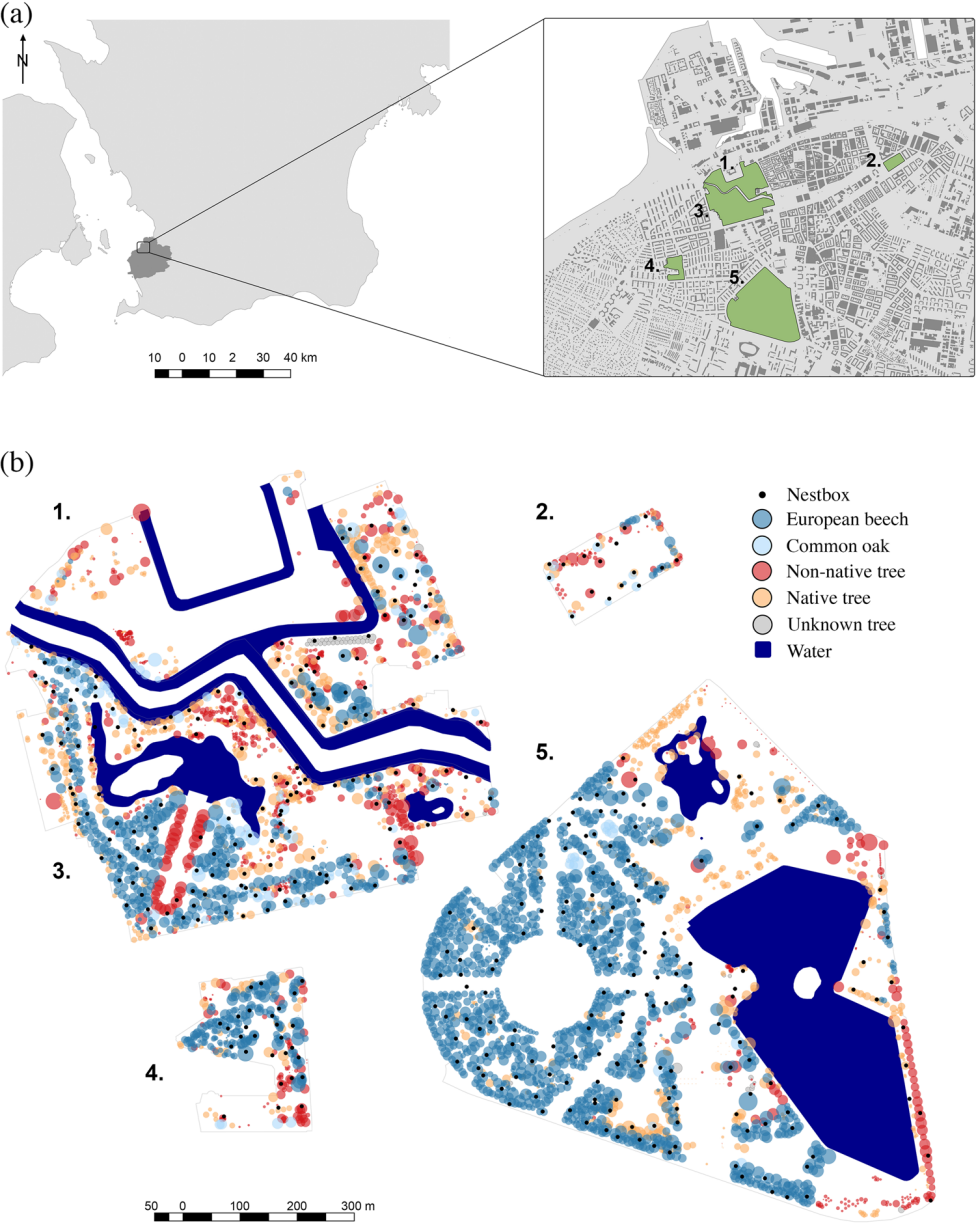
**a**, **b**. Study site and tree distribution. Maps of **a** the study site and parks within the city of Malmö and **b** the spatial distribution of nest boxes (black dots), European beech (*Fagus sylvatica*; dark blue circles), common oak (*Quercus robur*; light blue circles), non-native tree individuals (red circles), other native trees (not belonging to the species above, yellow circles) and trees of unknown species (grey circles) within parks (1–5). Circle size is proportional to the estimated canopy size. Note that, while parks in panel **b** are to scale and retaining the general orientation in relation to one another, the distance between the parks is not to scale for readability purposes. Parks 1 and 3 are only separated by a canal, approximately 20–30 m wide

We monitored nest box populations of great tits during the breeding season in 2013–2020 in 5 urban parks. The parks were located within the central area of the city of Malmö (55°35′24″ N 12°59′19″ E; Fig. 1a), which is the 3rd  largest city in Sweden with 350,000 inhabitants (SCB 2021). The parks ranged between 3 and 45 ha in size and housed a total of 400 nest boxes, spread evenly throughout them (Fig. 1b). The parks were characterized by a mixture of tree species, amenity grass, ponds and urban infrastructure such as paved roads, paths, lampposts and buildings. While great tits account for most breeding attempts in the nest box population, a significant number of blue tits also utilize the boxes, whose breeding season overlaps with that of great tits. A few observations of other passerine birds have also been recorded (see Appendix S2).

During the breeding season of great tits, which locally occurs April-June, the nest boxes were checked weekly. When eggs were found, the laying date was estimated by back-calculating from the number of eggs, as great tits typically lay one egg per day (Perrins 1970). When incubation was confirmed, the date of incubation start was calculated in a similar way, using the clutch size to estimate when incubation likely began. Nests were then left undisturbed for 12 days from the estimated incubation start and subsequently checked daily for hatching. Once nestlings reached 14 days from hatching, they were weighed and ringed. In 2016, not all nest boxes were monitored due to logistical constraints, and we, therefore, excluded the data from this year.

### Territory mapping

To investigate which aspects of the local tree composition affected the breeding success of urban great tits, we used the georeferenced tree database of Malmö, covering publicly managed land, as of August 2019 provided by the city of Malmö (pers. comm. Tim Delshammar). This database contains spatially explicit information on individual tree species, age, height, and crown radius. Using R packages *rgeos* and *sp* (Pebesma and Bivand 2005; Bivand et al. 2013; Bivand and Rundel 2020), we created a GIS polygon layer of the spatial coverage of all tree crowns by creating individual buffers of the crown radius for each tree based around the geographical position of the tree. Using a 35 m radius around each nest box, all tree canopies reaching within the radius were identified as part of the local tree composition. The 35 m cutoff point was chosen a priori as tits have been shown to hold territories within this area and tend, on average, to travel this distance to forage (Krebs 1971; Stauss et al. 2005; Jarrett et al. 2020).

We characterized urban territories based on a number of hypothesis-driven factors of the biotic environment: the number of common oak trees (*Quercus robur*), European beech trees (*Fagus sylvatica*), birch trees (*Betula pendula* and *B. pubescens*), tree diversity and number of non-native tree individuals. We included oak trees as they have been found to be a primary source of caterpillars and modulate the breeding phenology of great tits in forest populations (van Noordwijk et al. 1995; Visser et al. 2006). We included beech trees as this was by far the most common species within the parks (Jensen et al. 2022). Birch species were also common and are known to support relatively high caterpillar productivity (Eeva et al. 2000).

An inverse Simpson diversity index was included as we hypothesized that tree diversity could increase the number of potential different food sources within the territory (Ehrlich and Raven 1964). The abundance of non-native tree species was included as they host significantly fewer invertebrates compared with native trees (Jensen et al. 2022) and have been found to negatively affect avian reproduction (Narango et al. 2018). Non-native trees were defined as species introduced after the thirteenth century (Essl et al. 2018). While the tree composition could potentially change over the 8-year period the study was conducted, the average age of the trees located within the parks is over 80 years, and it is, therefore, unlikely that any major changes in the territories had occurred within the time-window of our study.

### Statistical analysis

All statistical analyses were conducted using R version 4.0.3 (R Core Team 2020). We constructed Generalized Linear Mixed Models (GLMMs), using the *glmmTMB* function from the *glmmTMB* package (Brooks et al. 2017), to analyze the correlation between local tree composition and breeding attempts, breeding onset, offspring survival and weight. We selected models through backward elimination while respecting marginality between interactive terms (see below; Zuur et al. 2007, 2009), where all models had the same initial structure where possible. We inspected model residuals manually, in addition to using the function *testResiduals* of the *DHARMa* package (Hartig 2020), to verify model assumptions (variance homogeneity and acceptable normality of model residuals).

To investigate which aspects of the tree composition influenced the likelihood of a breeding attempt, data from all boxes from the 7 years were used, totaling in a sample size of 2 800 cases. Note that we considered breeding attempts from both blue and great tits for this specific measure only, since not all breeding attempts could be confidently assigned to one of the two (e.g., if an observer could not identify nest ownership from tit eggs alone). Moreover, we expected interspecific competition for nest boxes, given the overlapping breeding seasons. A nest box was hence considered occupied when at least one blue or great tit egg was observed for the given year. We assigned occupied nest boxes the value 1 and unoccupied boxes 0. A nest box was considered unoccupied when no blue or great tit eggs were found. Note that for the few instances of other species breeding (*N* = 49, Appendix S2), the nest box was still assigned 0 since these breeding attempts generally occurred after the tit egg-laying period. We constructed an initial GLMM, with a logistic binomial distribution, with breeding attempt as the response variable and number of oaks, beech and birch trees, number of non-native trees, tree diversity, and year as fixed effects. The two-way interactions between year and all other main effects were also included. Neither lay date nor its interactions were included in the analysis on breeding attempts, as lay date could not be determined in all cases for this subset of data. The final GLMM, selected through backward elimination, included year, number of European beech trees and non-native tree individuals. Nest box was nested within park as a random structure, to account for the repeated sampling across years and potential differences between parks.

The response variable in the model for effects of local tree composition on breeding onset was the mean-centered laying date (day of first egg) per year for all great tit clutches that reached incubation, resulting in a sample size of 464 clutches. We only included eggs from completed clutches, as the species can be hard to determine up until this point, when the identity of the female is confirmed. The initial model had the same structure as above: number of oaks, beech and birch trees, number of non-native tree individuals, tree diversity, and year, together with the two-way interactions between year and all other fixed effects, were included. The final GLMM consisted of the number of common oak and European beech trees as fixed effects and nest box nested within park as a random factor. A Gaussian distribution was specified for this model. Inspection of model residuals revealed that variance was not homogenous between years in the model and the *dispformula* argument of the *glmmTMB* function was used to correct for the heteroscedastic variance (Brooks et al. 2017).

Great tit offspring survival, defined as the probability of an egg reaching day 14 as a nestling, was studied for all eggs of completed clutches where incubation onset was confirmed. In total, 3386 eggs from 464 clutches were included in the analysis. The initial model included number of oaks, beech and birch trees, number of non-native tree individuals, tree diversity, year and lay date (mean-centered per year) as fixed effects. The two-way interactions between year and lay date and all other factors were also included as fixed effects. The final GLMM included year, number of oak trees, tree diversity and lay date, together with the interactions between lay date and year, oak trees and lay date, and tree diversity and lay date. A random factor of clutch ID nested within park was included. A logistic binomial distribution was specified for the model.

To investigate the effect of local tree composition on nestling weight, the individual weight at day 14 of 1270 great tit nestlings, belonging to the 233 broods that reached this point in the 7 years, were included. The initial model had the same structure as above: number of oaks, beech and birch trees, number of non-native tree individuals, tree diversity, year and lay date (mean-centered per year) together with the two-way interactions between year and lay date and all other main effects. The final model, selected through backward elimination, included year, number of beech trees and non-native trees and lay date, together with the interactions between lay date and year, as well as beech trees and year, as fixed effects. A random factor of clutch ID nested within park was also included. A Gaussian distribution was used for the model. Inspection of model residuals revealed that variance was not homogenous between years in the model, and as above, the *dispformula* argument of the *glmmTMB* function was used to correct for the heteroscedastic variance (Brooks et al. 2017).

Since Park 5 had a significant proportion of the local non-native tree abundance located near the edge of the park, close to a road, we excluded the nest boxes along the park edge (13 nest boxes east of the main waterbody; Fig. 1b) from the models where non-native trees were included as a fixed effect (breeding occupancy and nestling weight). However, this potential spatial confounder did not alter the results qualitatively when excluded, and the full dataset was, therefore, used. In addition, note that there were no significant difference in age or size between native and non-native trees in the study system (Jensen et al. 2022). Clutch size was not included in models for nestling weight or offspring survival; all significant results were, however, retained for both final models when we added clutch size to control this did not influence the results. To control for co-linearity between factors, variance inflation factors (VIFs) were calculated for all initial models, excluding interactions, using the *check_collinearity* function of the *performance* package (Lüdecke et al. 2020). No VIFs > 2 were found (Appendix S3), indicating negligible co-linearity between fixed effects (Zuur et al. 2007). Models were compared with the *anova* function in the backward elimination process (Appendix S4). *P* values were obtained with function *Anova* (*car* package) on final models using Type III Wald chi-square tests. *R*^2^ values were calculated through the *r2* function of the *performance* package.

## Results

**Fig. 2:**
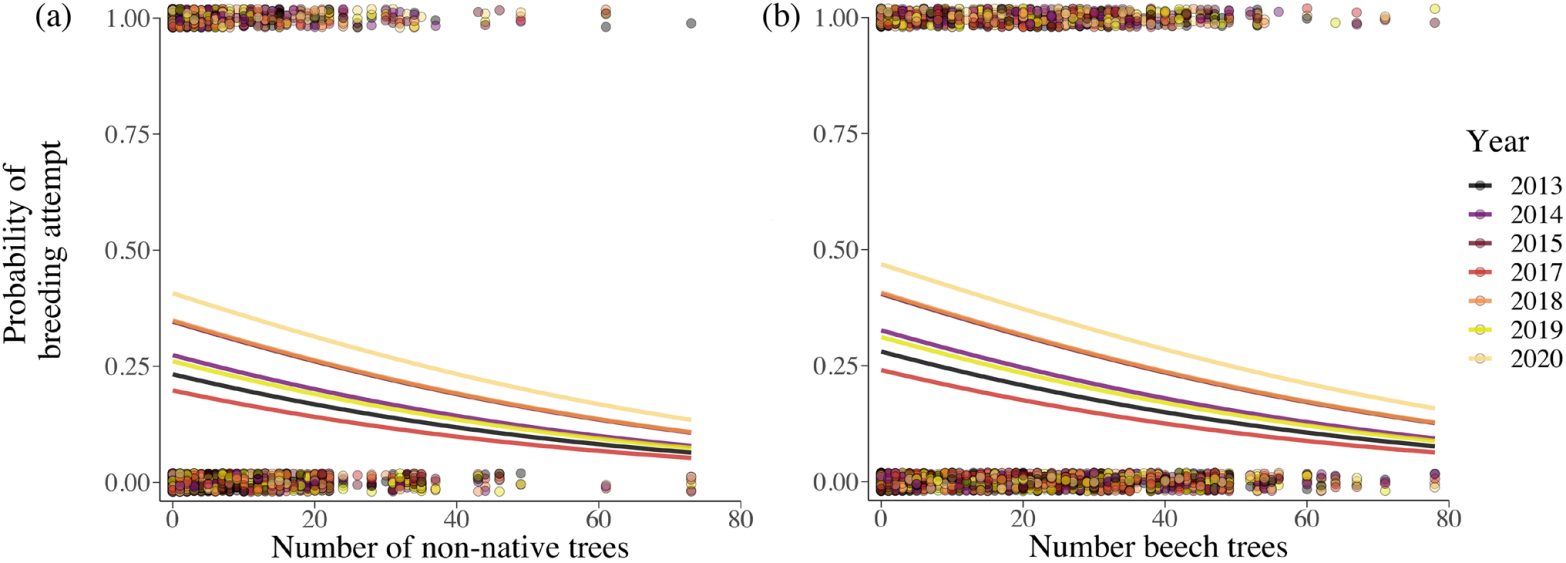
**a**, **b**. Breeding attempt rate depending on local tree composition and year. The probability of urban blue and great tit breeding attempts showed negative relationships with the number of **a** non-native tree individuals and **b** European beech trees (*Fagus sylvatica*) across all years. Colors denote year

The total number of blue and great tit breeding attempts in the nest box population varied significantly between the years (Table 1). The likelihood of a breeding attempt was negatively correlated to numbers of European beech trees and non-native trees within the territory. Thus, nest boxes located in territories with high abundance of either of these trees were less likely to be occupied by birds during the breeding season (Fig. 2a, b, Table 1). The onset of breeding in great tits (i.e., the mean-centered lay date of the first egg) showed a significant and positive correlation with the abundance of common oak trees and European beech trees within the territory. Specifically, great tits in oak- and beech-rich territories started breeding later than the year average (Table 1).

**Table 1:** Results of statistical analyses

Response variable	Fixed effects	χ^2^	*P* value
Breeding attempt (1/0)	**Year**	58.054	** < 0.001**
*N* = 2800 (400)	**Beech trees**	19.395	** < 0.001**
*R*^2^*c* = 0.20	**Non-native trees**	9.273	**0.002**
*R*^2^*m* = 0.05			
Lay date (days)	**Oak trees**	16.753	** < 0.001**
*N* = 464 (276)	**Beech trees**	5.872	**0.015**
*R*^2^*c* = 0.14			
*R*^2^*m* = 0.04			
Offspring survival	**Lay date**	13.617	** < 0.001**
(1/0)	**Year**	22.705	** < 0.001**
*N* = 3 386 (276)	Oak trees	3.204	0.073
*R*^2^*c* = 0.88	Tree diversity	2.064	0.151
*R*^2^*m* = 0.13	**Lay date × year**	13.398	**0.037**
	**Lay date × tree diversity**	7.431	**0.006**
	**Lay date × oak trees**	5.897	**0.015**
Nestling weight (g)	**Lay date**	25.204	** < 0.001**
*N* = 1270 (176)	Year	9.048	0.171
*R*^2^*c* = 0.73	Beech trees	0.279	0.597
*R*^2^*m* = 0.23	**Non-native trees**	8.128	**0.004**
	**Year × lay date**	25.530	** < 0.001**
	**Year × beech trees**	13.908	**0.031**

Outputs of statistical models, including final GLMMs selected by backward elimination, on breeding attempts, breeding onset, offspring survival and weight (see Table S5 for effect sizes). Lay date was in all cases mean-centered per year. Significant results are typed in bold. *N* denotes sample size and numbers in parentheses denote the number of unique territory profiles included in each model. *R*^2^*c* denotes the conditional *R*^2^ value, which takes fixed and random effects into account (nest box nested within park for models on breeding attempts and lay date; clutch ID nested within park for offspring survival and weight; see Methods) and *R*^2^*m* denotes the marginal *R*^2^ value, which only assess the fixed effects

**Fig. 3:**
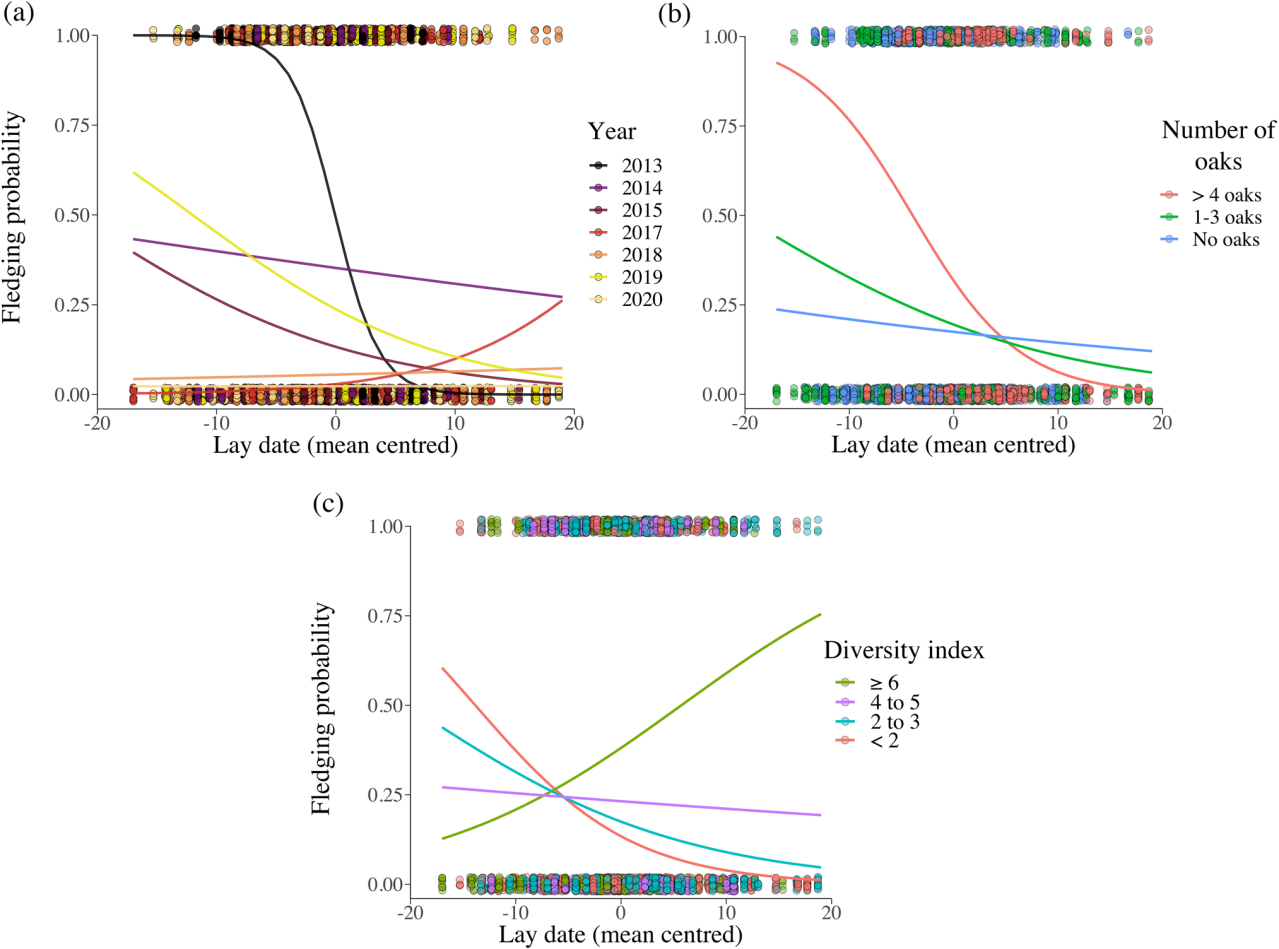
**a**–**c** Likelihood of great tit eggs reaching fledging**.** A graphical representation of the significant interactions modulating offspring survival between mean-centered lay date and **a** year, **b** the number of common oak trees (*Quercus robur*) and **c** the inverse Simpson index of tree diversity within the territory. Lay date is mean-centered per year and the Simpson index is inverse, meaning that higher values equate to higher diversity (see Methods for details). Although modeled as covariates, number of oaks and diversity index are separated into discrete groups for ease of visualization and corresponding trendlines represent the average model fit within the groups (denoted by color)

Great tit offspring survival was significantly correlated with several interactions between the onset of breeding and other factors. First, the effect of timing of breeding on offspring survival varied significantly in strength and direction between years (significant interaction between lay date and year; Fig. 3a, Appendix S5). In most years, a higher probability of survival was associated with early breeding, but the overall survival probability varied greatly between years. Second, offspring survival was significantly affected by interactions between the number of common oak trees and within-year onset of breeding (Fig. 3b), as well as between the inverse Simpson index of tree diversity and within-year onset of breeding (Fig. 3c). Earlier onset of breeding correlated with a higher likelihood of offspring survival in oak-rich territories compared to territories with no, or fewer, oak trees (Fig. 3b), whereas in territories with very high tree diversity, later onset of breeding was associated with higher offspring survival compared with territories characterized by moderate to low tree diversity (Fig. 3c).

**Fig. 4:**
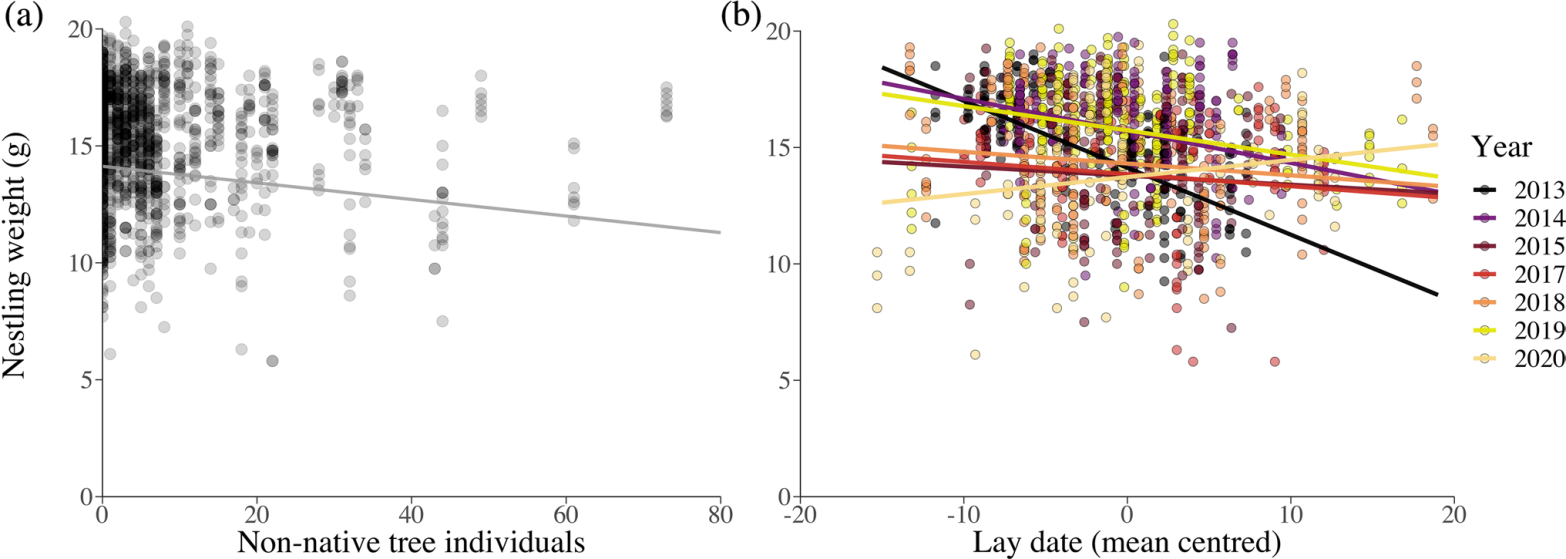
**a**, **b** Nestling weight depending on local tree composition and breeding onset. Plots visualizing the significant effects on urban great tit nestling weight of **a** the number of non-native trees within the territory and **b** the significant interaction between mean-centered lay date and year. Colors in **b** denote year

Furthermore, nestling weight was significantly lower when there were more non-native trees in the territory (Fig. 4a). In addition, breeding earlier or later within a year had different effects on nestling weight depending on year (Fig. 4b; Appendix S5). In most years, breeding early was associated with heavier chicks, while in other years, the association was less pronounced. Finally, the number of European beech trees within a territory showed significant interactions with year on nestling weight, varying annually in terms of the direction of the effect.

## Discussion

We used high-resolution spatial data to investigate how tree composition of urban territories relates to the number of breeding attempts by blue and great tits, and the breeding onset and reproductive output of great tits. Using a long-term dataset of 400 nest boxes in 5 parks within a city over 7 years, we found that an increasing number of non-native trees in a territory had a consistently negative relationship with the likelihood of blue and great tit breeding attempts and on great tit nestling weight, across all years. However, the onset of great tit breeding and offspring survival was not affected by the number of non-native trees. While offspring survival (i.e., short-term survival) was not directly affected by non-native trees in the territory, body mass at fledging is typically strongly positively linked to post-fledging survival in birds (Naef-Daenzer et al. 2001). Thus, great tits reared in territories dominated by native trees are more likely to survive and recruit into the population. In addition, we found that great tit offspring survival and nestling weight were more strongly associated with interactive effects between study-year and onset of breeding, compared with the local tree composition per se, which in turn affected these two measures of breeding success in different ways.

### Avoidance of non-native trees

We found that the likelihood of blue and great tit breeding attempts and great tit nestling weight significantly decreased with increasing numbers of non-native tree individuals (Fig. 2a). Importantly, our results suggest, together with previous studies (Narango et al. 2018), that the effect of non-native trees is consistently negative across avian species, geographical regions and habitat types, including urban parks where non-native trees may be especially favored by city-planners. Our results are likely explained by the fact that non-native plants host less abundant and diverse invertebrate assemblages, which would be expected given a lack of co-evolutionary history between plants and herbivores (Berthon et al. 2021). Analyzing effects of non-native trees has previously been somewhat overlooked in the few detailed studies on food availability to birds in urban systems (e.g., Jarrett et al. 2020; Pollock et al. 2017; Seress et al. 2018, but see Narango et al. 2018), especially at a tree species-level resolution. In our study system, non-native tree individuals account for almost 25% of the total tree abundance (Jensen et al. 2022). Notably, our results show that, although non-native trees represent a minority, they have a consistently negative relationship with avian reproduction across years in city-center parks, highlighting the importance of native trees in cities.

Although we found no co-linearity between tree diversity and number of non-native trees, it is worth noting that non-native species constitute the clear majority of tree species richness in the study system (Jensen et al. 2022). Thus, territories with the highest tree diversity would likely also have a significant proportion of non-native tree canopy (Appendix S6). We believe this at least partly explains why late breeding increased great tit offspring survival probability in territories with a highly diverse tree composition (Fig. 3c). Non-native trees have a delayed phenology compared to local native species in the current study system (Jensen et al. 2022), and it is, therefore, likely that breeding later in territories with non-native trees could ensure higher food availability. In addition, high tree diversity could result in a multitude of local food peaks and a more homogenous food availability along the breeding season, allowing birds to avoid detrimental cold snaps by breeding later without food limitation.

Our results suggest that urban tits can distinguish and discriminate between vegetational composition types early in the breeding season, demonstrated by their avoidance of relatively low-quality territory components such as a high abundance of non-native trees. However, as no direct association with great tit offspring survival could be attributed to the abundance of non-native trees, it is likely that at least great tits are able to compensate for the lower territory quality through higher foraging effort or by reliance on less favored prey items to a certain extent (Narango et al. 2018; Jarrett et al. 2020). It is also possible that birds are able to mitigate some of the negative effects of non-native trees by avoiding territories within areas with high percentage of non-native trees. Indeed, a linear model performed post hoc showed that the proportion of occupied territories with above-average numbers of non-native trees increased in line with the annual bird population size (total number of annual breeding attempts; *P* < 0.001). This suggests that more birds occupy territories rich in non-native trees when competition increases, likely being pushed out from the more attractive territories (with high native tree density) by more competitive individuals (Appendix S7). However, while non-native tree species generally support less diverse assemblages at higher trophic levels, large variation is also found among native plant species (Narango et al. 2020). Thus, although tree species’ origin could potentially serve as a simple indicator of habitat quality in urban ecosystem conservation (Jensen et al. 2022), identifying species-specific contributions of different trees in supporting invertebrate food sources to birds would be needed to gain a deeper understanding of key trophic interactions in urban systems.

### Temporal variation of oak and beech tree effects

The delayed within-year onset of breeding in territories with high numbers of oak trees was expected as great tits have been found to modulate their breeding following the phenology of the trees and the subsequent food source of caterpillars (van Noordwijk et al. 1995). While variation between years in onset of breeding is expected to be mainly influenced by temperature (van Noordwijk et al. 1995), breeding relatively early or late within a year may reflect an adaptive response by birds to the local tree composition and food availability (Nilsson and Källander 2006; Visser et al. 2006). Indeed, the phenology of common oak trees and the peak of caterpillar biomass hosted by oaks is later than other local native tree species (Jensen et al. 2022, also see Appendix S1) and could, therefore, potentially cue great tits to this response.

Surprisingly, however, breeding earlier in oak-rich territories was associated with a higher chance of great tit offspring survival, although increasing numbers of oak trees were also associated with delayed reproduction in terms of lay date. These results indicate that urban birds may be mismatched to the phenology of the local food peaks; the oak trees appear to cause later breeding, yet earlier clutches had a higher chance of survival in these territories. Such potential phenological mismatches have previously been linked to, e.g., climate change (Both et al. 2009), but has also been suggested to occur in urban environments (Fisogni et al. 2020). The factors causing variation in breeding onset and the implications of breeding onset itself are discussed further below.

In contrast to oaks, the likelihood of breeding attempts in territories decreased with increasing numbers of European beech trees (Fig. 2b), most likely because beeches provide relatively few caterpillars (Jensen et al. 2022). Beech trees were highly abundant in the studied parks, and therefore, many nest boxes were situated in beech-dominated, homogenous territories, with few additional tree species providing supplementary food sources. The observed finding that great tits bred later when European beech trees were more abundant in the territory may appear unexpected, given that this tree species is unlikely to modulate the reproduction of great tits, due to its low insect abundance during the breeding period (Jensen et al. 2022). However, it is possible that the later within-year onset of breeding in beech-rich habitats is explained by other territories becoming occupied earlier in the season. Thus, later breeders may not have a wide choice of territories and, consequently, become over-represented in beech-rich territories. In addition, the correlation between nestling weight and the local abundance of European beech trees varied across years. This result indicates that the food sources that beech trees provide differ between years, potentially depending on the general onset of spring and the ability of the birds to take advantage of resource pulses. Alternatively, years of larger great tit population sizes and higher occupancy of territories could force high-quality birds to breed in the poorer beech-dominated territories, similar to what we observed for non-native trees.

### Breeding onset and annual variation

We found significant annual variation in blue and great tit breeding attempts in the nest boxes, which is not surprising as the competition for high-quality territories likely varies with fluctuations in population size, due to, e.g., winter survival (Balen 1980). Offspring survival of great tits was significantly affected by several interactions involving the onset of breeding. Notably, the strength of the association between survival and onset of breeding varied markedly between years (Fig. 4b), which is perhaps surprising given that avian populations generally tend to be under selection to advance egg laying (Shipley et al. 2020; Villemereuil et al. 2020)*.* Earlier onset of breeding has been predicted and observed in response to climate change (Visser et al. 2006; Shipley et al. 2020) but has also been suggested as a general adaptive response in great tits to better match local food sources, since delaying reproduction is more feasible than speeding it up (van Noordwijk et al. 1995). As such, one could expect urban birds to show strong selection for earlier breeding, especially since factors such as the urban heat island effect tend to advance urban plant phenology (Li et al. 2019, but see Vaugoyeau et al. 2016). However, the urban environment is complex and as our results show, local tree composition can interact with breeding onset and affect offspring survival (Fig. 3a–c). Indeed, earlier onset of breeding may not be uniformly expected in all contexts and can depend on food availability (Svensson and Nilsson 1995; Shutt et al. 2021). Furthermore, a risk of early onset of reproduction is the increased probability of cold snaps, which can be detrimental to insects, and in turn, insectivorous birds (Shipley et al. 2020). Indeed, a recent meta-analysis has confirmed that the increased variation in breeding phenology of urban birds is a general pattern (Capilla-Lasheras et al. 2022).

Heterogeneous between-year variation in reproductive traits in our study could be explained by a physiological constraint on breeding early in years with low food abundance through nutritional limitation of females. This could act in synergy with cold snaps, which can both reduce food availability and increase chick mortality (Shipley et al. 2020). Thus, the inter-annual variation in the correlation between onset of breeding and offspring survival could be explained by either food limitation delaying breeding in years with low food abundance, and/or occasional cold snaps taking a toll on early clutches. It is worth noting that the breeding season of 2013, showing the strongest negative relationship between survival and breeding onset, also had (i) the coldest mid-March to mid-April temperatures in the dataset (based on temperature data provided by ECA&D, Cornes et al. 2018) and (ii) the latest average lay dates. However, more research needs to be carried out to fully understand the mechanisms behind the observed pattern. While the direction of the relationship varies between years, overall, breeding relatively early resulted in higher offspring survival, suggesting a general selection for early breeding.

### Limitations and future studies

Tree composition offers a link between the structure of managed urban green spaces and the hypothesis of food limitation as a major driver of the reduced breeding success often observed in urban birds. Explicitly unaccounted variation caused by abiotic factors may also play an important part, and conditional *R*^2^ values, which take random terms into account, were markedly higher than marginal *R*^2^ values, which only assess the fixed effects, for all models (Table 1). As the random structure included individual nest box or clutch identity, the conditional *R*^2^ value implicitly accounts for a variety of local habitat effects. Thus, the discrepancy between the two could arise from the exclusion of abiotic factors such as artificial light at night, proximity to roads, air pollution and other sources of disturbance (Remacha and Delgado 2009; Dominoni et al. 2020; Corsini et al. 2021; Plummer et al. 2021). However, large variation is also expected, and likely explained, by the 35 m radius used to define the extent of the foraging areas surrounding each nest box. Although this choice was made a priori based on earlier research (Jarrett et al. 2020), we acknowledge that birds are likely to be affected by biotic factors—including tree composition—outside this radius, especially in lower quality territories where they might travel farther to forage (Jarrett et al. 2020). The *R*^2^ values also indicate that the fixed effect components of the models for nestling weight and survival explain a large majority of the variance, while the models for breeding attempts and breeding onset explain less. This suggests that factors other than habitat quality cause the majority of the observed variance in probability of breeding and onset of breeding, which instead could be governed by, e.g., weather, population size and parental fitness (Svensson and Nilsson 1995; Nussey et al. 2005).

We did not differentiate between blue and great tits in our analyses of probability of breeding attempts and timing of breeding, partly because of varying quality of species identity data based on eggs (see Methods), but mainly as interspecific competition for territories is to be expected. Great and blue tits were by far the most common breeders in our nest box population (~ 95%; Appendix S2), and while these species are similar in their breeding behavior, some differences in their diet and foraging could be expected (Betts 1955). While accounting for interspecific competition could resolve some unexplained variation in our analysis, the strength of this effect is most likely dependent on complicated biotic and abiotic interactions, which are beyond the scope of this study. Hence, we acknowledge that the specificity of our model on breeding attempts is probably lower compared to the other models, where great tits alone were studied. Nevertheless, our results show that the negative effects of non-native trees and beech trees are strong enough to be detected across the two species. Future research is needed to determine whether birds are affected by interspecific competition and limited by the amount or quality of food, and to which extent these factors are regulated by tree composition or other effects in the urban environment, such as air pollution and artificial light at night.

### Conclusions

Using a unique, long-term dataset on the relationship between avian reproductive traits and tree composition of the urban habitat on a high-resolution spatial scale, our study offers two main conclusions. First, we show that urban birds avoid territories dominated by non-native trees during the breeding season and that nestling weight decreases with an increasing number of non-native trees. From a conservation perspective, specific native species known to host invertebrates, such as oaks (*Quercus spp.*), should be prioritized over and above non-native trees in management decisions regarding urban green spaces. However, we found that common oak may cue urban great tits to delay their egg laying; this response does not appear to be adaptive as offspring survival probability was increased by breeding earlier, specifically in oak-dominated territories. Taken together, this could indicate a mismatch to local food sources and, therefore, planting native trees might not be sufficient on its own, but should be done in concert with interventions aiming to decrease the risk of phenological mismatches, e.g., decreasing the urban heat island effect. Second, we show that the urban tree composition has strong variation in its association with nesting success of an insectivorous bird, both within and between years. Temporal variation most likely reflects variation in weather and food sources that cause contrasting responses in the reproductive success of great tits among territories. We, therefore, conclude that urban habitat quality for breeding birds is variable and hard to predict due to temporal variation, yet the negative impacts of non-native vegetation are consistent across years.

## Supplementary Information

Below is the link to the electronic supplementary material.

Supplementary file1 (DOCX 268 kb)

## Data Availability

Data will be archived in Figshare upon acceptance of the manuscript.
