# Supplementary material for: Urban tree composition is associated with breeding success of a passerine bird, but effects vary within and between years
Source: Oecologia. 2023 Jan 21;201(3):585–97. doi: 10.1007/s00442-023-05319-8 (PMC10038977; doi:10.1007/s00442-023-05319-8)
Supplement: Supplementary file 1 — Supplementary file1 (DOCX 268 kb) [file 442_2023_5319_MOESM1_ESM.docx]

**Urban tree composition is associated with breeding success of a passerine bird, but effects vary within and between years**

Johan Kjellberg Jensen^1,2*^, Johan Ekroos^2,3^, Hannah Watson^1^, Pablo Salmón^4^, Peter Olsson^2^, Caroline Isaksson^1^

^1^ Department of Biology, Lund University, Lund, Sweden

^2^ Centre for Environmental and Climate Science (CEC), Lund University, Lund, Sweden

^3^ Department of agricultural sciences, University of Helsinki, Helsinki, Finland

^4^ Institute of Biodiversity, Animal Health and Comparative Medicine, University of Glasgow, Glasgow, UK

* Correspondence to: Johan Kjellberg Jensen, Lund University, Sweden

E-mail: [johan.kjellberg_jensen@biol.lu.se](mailto:johan.kjellberg_jensen@biol.lu.se)

APPENDIX

**Table S1. Bud burst phenology depending on tree species and origin in the system.** Bud burst dates of locally common tree species, recorded in 2019. Bold denotes focal species of the current study. Adapted from Jensen et al. (2022), see original publication for details.

| **Species** | **Origin** | **N** | **Mean bud burst (days from March 31st)** | | **Standard error (SE)** |
| --- | --- | --- | --- | --- | --- |
| *Acer platanoides* | Native | 6 | 29 | 1.00 | |
| ***Betula pendula*** | Native | 5 | 21.6 | 1.99 | |
| *Crataegus monogyna* | Native | 4 | 24 | 1.22 | |
| ***Fagus sylvatica*** | Native | 5 | 25.2 | 1.20 | |
| *Prunus avium* | Native | 5 | 23.4 | 1.12 | |
| ***Quercus robur*** | Native | 5 | 30 | 1.34 | |
| *Salix caprea* | Native | 4 | 20.25 | 1.44 | |
| *Sorbus intermedia* | Native | 1 | 12 | NA | |
| *Tilia x europaea* | Native | 5 | 25.8 | 0.73 | |
| *Ailanthus altissima* | Nonnative | 4 | 33.75 | 1.44 | |
| *Ginkgo biloba* | Nonnative | 5 | 28.2 | 2.24 | |
| *Gleditsia triacanthos* | Nonnative | 4 | 34.5 | 1.50 | |
| *Platanus x hispanica* | Nonnative | 5 | 37.2 | 1.20 | |
| *Pterocarya fraxinifolia* | Nonnative | 5 | 29.4 | 1.12 | |
| *Quercus cerris* | Nonnative | 5 | 30 | 0.00 | |
| *Robinia pseudoacacia* | Nonnative | 5 | 32.4 | 0.60 | |
| Native average |  | 40 | 24.83 | 0.72 | |
| Nonnative average |  | 33 | 32.09 | 0.70 | |
|  |  |  |  | |  |


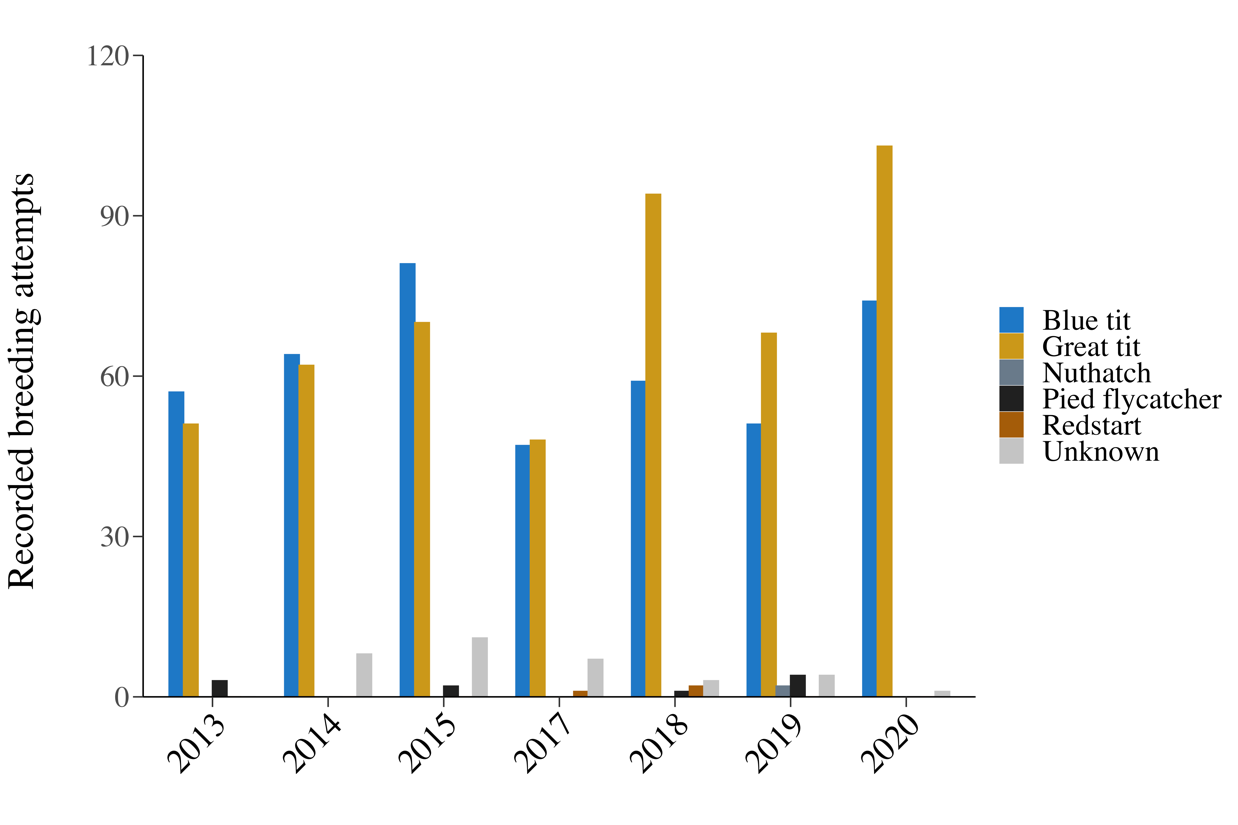


***Figure S2.* Distribution of breeding attempts by species over years.** The absolute number of breeding attempts (defined as an egg being laid; see Methods) within the population varied between years and was dominated by great tits (*Parus major*) and blue tits (*Cyanistes caeruleus*). A low number of nuthatch (*Sitta europaea*), pied flycatcher (*Ficedula hypoleuca*) and redstart (*Phoenicurus phoenicurus*) breeding attempts were also recorded. In certain cases, species could not be reliably determined; note however that these nests likely belonged to either great or blue tits. Color denotes species.

***Table S3.* Variance inflation factors (VIFs) of initial models.** Output from the inspection of potential co-linearity between fixed effects for initial models. VIFs below > 2 indicate low co-linearity.

| **Model** | **Fixed effects** | **VIF** | **Increased SE** | **Tolerance** |
| --- | --- | --- | --- | --- |
| *Breeding attempt (1/0)* | Year | 1.00 | 1.00 | 1.00 |
|  | Birch trees | 1.04 | 1.02 | 0.97 |
|  | Oak trees | 1.03 | 1.01 | 0.97 |
|  | Beech trees | 1.30 | 1.14 | 0.77 |
|  | Tree diversity | 1.45 | 1.20 | 0.69 |
|  | Non-native trees | 1.41 | 1.19 | 0.71 |
| *Lay date (days)* | Year | 1.04 | 1.02 | 0.97 |
|  | Birch trees | 1.08 | 1.04 | 0.92 |
|  | Oak trees | 1.04 | 1.02 | 0.96 |
|  | Beech trees | 1.70 | 1.30 | 0.59 |
|  | Tree diversity | 1.90 | 1.38 | 0.53 |
|  | Non-native trees | 1.38 | 1.18 | 0.72 |
| *Offspring survival* | Lay date | 1.05 | 1.02 | 0.96 |
|  | Year | 1.03 | 1.02 | 0.97 |
|  | Birch trees | 1.09 | 1.05 | 0.91 |
|  | Oak trees | 1.09 | 1.04 | 0.92 |
|  | Beech trees | 1.71 | 1.31 | 0.58 |
|  | Tree diversity | 1.88 | 1.37 | 0.53 |
|  | Non-native trees | 1.40 | 1.18 | 0.71 |
| *Nestling weight (g)* | Lay date | 1.05 | 1.03 | 0.95 |
|  | Year | 1.10 | 1.05 | 0.91 |
|  | Birch trees | 1.12 | 1.06 | 0.90 |
|  | Oak trees | 1.08 | 1.04 | 0.92 |
|  | Beech trees | 1.62 | 1.27 | 0.62 |
|  | Tree diversity | 1.65 | 1.28 | 0.61 |
|  | Non-native trees | 1.41 | 1.19 | 0.71 |

***Table S4.* Statistical output from the elimination process.** Output from the backward elimination process for all models, sorted in the order in which each variable was removed.

| **Model** | **Fixed effects** | **Eliminated** | **χ^2^** | **df** | **p-value** |
| --- | --- | --- | --- | --- | --- |
| *Breeding attempt (1/0)* | Non-native trees x Year | 1 | 1.583 | 6 | 0.954 |
|  | Birch trees x Year | 2 | 1.622 | 6 | 0.951 |
|  | Oak trees x Year | 3 | 3.594 | 6 | 0.731 |
|  | Tree diversity x Year | 4 | 7.126 | 6 | 0.309 |
|  | Beech trees x Year | 5 | 9.369 | 6 | 0.154 |
|  | Tree diversity | 6 | 0.025 | 6 | 0.873 |
|  | Oak trees | 7 | 1.708 | 6 | 0.191 |
|  | Birch trees | 8 | 1.465 | 6 | 0.226 |
| *Lay date (days)* | Oak trees × Year | 1 | 1.695 | 6 | 0.946 |
|  | Birch trees × Year | 2 | 4.089 | 6 | 0.665 |
|  | Beech trees × Year | 3 | 7.206 | 6 | 0.302 |
|  | Tree diversity × Year | 4 | 4.437 | 6 | 0.618 |
|  | Non-native trees × Year | 5 | 6.284 | 6 | 0.392 |
|  | Year | 6 | 0.207 | 6 | 1.000 |
|  | Non-native trees | 7 | 0.212 | 1 | 0.645 |
|  | Birch trees | 8 | 0.199 | 1 | 0.656 |
|  | Tree diversity | 9 | 0.359 | 1 | 0.549 |
| *Offspring survival* | Non-native trees × Year | 1 | 0.284 | 1 | 0.594 |
|  | Birch × Lay date | 2 | 0.024 | 1 | 0.876 |
|  | Beech trees × Lay date | 3 | 0.318 | 1 | 0.573 |
|  | Non-native trees × Lay date | 4 | 0.711 | 1 | 0.399 |
|  | Oak trees × Year | 5 | 7.985 | 6 | 0.239 |
|  | Beech × Year | 6 | 10.766 | 6 | 0.096 |
|  | Simpson × Year | 7 | 6.782 | 6 | 0.341 |
|  | Birch × Year | 8 | 8.427 | 6 | 0.208 |
|  | Beech trees | 9 | 0.041 | 1 | 0.840 |
|  | Non-native trees | 10 | 1.705 | 1 | 0.192 |
|  | Birch trees | 11 | 3.330 | 1 | 0.068 |
| *Nestling weight (g)* | Tree diversity x Lay date | 1 | 0.158 | 1 | 0.691 |
|  | Beech trees x Lay date | 2 | < 0.001 | 1 | 0.990 |
|  | Tree diversity x Year | 3 | 2.857 | 6 | 0.827 |
|  | Non-native trees x Lay date | 4 | 1.293 | 1 | 0.256 |
|  | Birch trees x Lay date | 5 | 1.406 | 1 | 0.236 |
|  | Oak trees x Year | 6 | 9.579 | 6 | 0.144 |
|  | Non-native trees x Year | 7 | 8.990 | 6 | 0.174 |
|  | Birch trees x Year | 8 | 9.316 | 6 | 0.157 |
|  | Oak trees x Lay date | 9 | 3.300 | 1 | 0.069 |
|  | Tree diversity | 10 | 0.156 | 1 | 0.693 |
|  | Oak trees | 11 | 0.217 | 1 | 0.641 |
|  | Birch trees | 12 | 1.003 | 1 | 0.317 |

***Table S5.* Model trends from year × lay date interactions.** Output of function *emtrends* function of the *emmeans* package (Lenth 2021) for ***(a)*** lay date trends of individual years, tested against zero and ***(b)*** pairwise comparison of lay date trends between years. Significant results are typed in bold.

| a) |  |  |  | |  |  |  |
| --- | --- | --- | --- | --- | --- | --- | --- |
| **Model** | **Year** | **Lay date trend** | | **SE** | **df** | **t-ratio** | **p-value** |
| *Offspring survival* | **2013** | **-0.538** | **0.156** | | **3366** | **-3.450** | **0.001** |
| *(1/0)* | 2014 | -0.020 | 0.155 | | 3366 | -0.128 | 0.898 |
|  | 2015 | -0.085 | 0.130 | | 3366 | -0.656 | 0.512 |
|  | 2017 | 0.130 | 0.111 | | 3366 | 1.170 | 0.242 |
|  | 2018 | 0.016 | 0.074 | | 3366 | 0.211 | 0.833 |
|  | 2019 | -0.097 | 0.112 | | 3366 | -0.864 | 0.388 |
|  | 2020 | 0.001 | 0.079 | | 3366 | 0.008 | 0.994 |
| *Nestling weight (g)* | **2013** | **-0.288** | **0.057** | | **1239** | **-5.020** | **< 0.001** |
|  | **2014** | **-0.138** | **0.066** | | **1239** | **-2.096** | **0.036** |
|  | 2015 | -0.039 | 0.063 | | 1239 | -0.615 | 0.539 |
|  | 2017 | -0.052 | 0.066 | | 1239 | -0.792 | 0.428 |
|  | 2018 | -0.051 | 0.037 | | 1239 | -1.384 | 0.167 |
|  | **2019** | **-0.104** | **0.050** | | **1239** | **-2.099** | **0.036** |
|  | 2020 | 0.073 | 0.047 | | 1239 | 1.551 | 0.121 |

| b) |  |  |  |  |  |  |
| --- | --- | --- | --- | --- | --- | --- |
| **Model** | **Contrast** | **Estimate** | **SE** | **df** | **t-ratio** | **p-value** |
| *Offspring survival* | 2013-2014 | -0.518 | 0.218 | 3366 | -2.372 | 0.211 |
| *(1/0)* | 2013-2015 | -0.452 | 0.200 | 3366 | -2.267 | 0.261 |
|  | **2013-2017** | **-0.668** | **0.192** | **3366** | **-3.484** | **0.009** |
|  | **2013-2018** | **-0.553** | **0.175** | **3366** | **-3.170** | **0.026** |
|  | 2013-2019 | -0.441 | 0.190 | 3366 | -2.321 | 0.234 |
|  | **2013-2020** | **-0.539** | **0.175** | **3366** | **-3.075** | **0.035** |
|  | 2014-2015 | 0.066 | 0.201 | 3366 | 0.326 | 1.000 |
|  | 2014-2017 | -0.150 | 0.191 | 3366 | -0.786 | 0.986 |
|  | 2014-2018 | -0.035 | 0.173 | 3366 | -0.205 | 1 |
|  | 2014-2019 | 0.077 | 0.192 | 3366 | 0.402 | 1.000 |
|  | 2014-2020 | -0.021 | 0.175 | 3366 | -0.118 | 1 |
|  | 2015-2017 | -0.216 | 0.170 | 3366 | -1.265 | 0.868 |
|  | 2015-2018 | -0.101 | 0.151 | 3366 | -0.669 | 0.994 |
|  | 2015-2019 | 0.012 | 0.173 | 3366 | 0.067 | 1 |
|  | 2015-2020 | -0.086 | 0.154 | 3366 | -0.561 | 0.998 |
|  | 2017-2018 | 0.115 | 0.132 | 3366 | 0.867 | 0.977 |
|  | 2017-2019 | 0.227 | 0.158 | 3366 | 1.437 | 0.782 |
|  | 2017-2020 | 0.130 | 0.136 | 3366 | 0.950 | 0.964 |
|  | 2018-2019 | 0.113 | 0.134 | 3366 | 0.842 | 0.981 |
|  | 2018-2020 | 0.015 | 0.106 | 3366 | 0.140 | 1 |
|  | 2019-2020 | -0.098 | 0.136 | 3366 | -0.718 | 0.992 |
| *Nestling weight (g)* | 2013-2014 | -0.150 | 0.087 | 1239 | -1.732 | 0.595 |
|  | 2013-2015 | -0.248 | 0.086 | 1239 | -2.907 | 0.057 |
|  | 2013-2017 | -0.235 | 0.087 | 1239 | -2.698 | 0.100 |
|  | **2013-2018** | **-0.237** | **0.067** | **1239** | **-3.540** | **0.008** |
|  | 2013-2019 | -0.183 | 0.075 | 1239 | -2.449 | 0.179 |
|  | **2013-2020** | **-0.361** | **0.075** | **1239** | **-4.839** | **< 0.001** |
|  | 2014-2015 | -0.099 | 0.091 | 1239 | -1.084 | 0.933 |
|  | 2014-2017 | -0.086 | 0.093 | 1239 | -0.924 | 0.969 |
|  | 2014-2018 | -0.087 | 0.075 | 1239 | -1.171 | 0.905 |
|  | 2014-2019 | -0.033 | 0.082 | 1239 | -0.408 | 1.000 |
|  | 2014-2020 | -0.211 | 0.081 | 1239 | -2.601 | 0.127 |
|  | 2015-2017 | 0.013 | 0.091 | 1239 | 0.143 | 1.000 |
|  | 2015-2018 | 0.011 | 0.072 | 1239 | 0.159 | 1.000 |
|  | 2015-2019 | 0.065 | 0.079 | 1239 | 0.825 | 0.982 |
|  | 2015-2020 | -0.112 | 0.080 | 1239 | -1.399 | 0.803 |
|  | 2017-2018 | -0.002 | 0.075 | 1239 | -0.020 | 1.000 |
|  | 2017-2019 | 0.052 | 0.083 | 1239 | 0.633 | 0.996 |
|  | 2017-2020 | -0.125 | 0.081 | 1239 | -1.545 | 0.717 |
|  | 2018-2019 | 0.054 | 0.061 | 1239 | 0.885 | 0.975 |
|  | 2018-2020 | -0.124 | 0.060 | 1239 | -2.054 | 0.381 |
|  | 2019-2020 | -0.178 | 0.070 | 1239 | -2.552 | 0.142 |


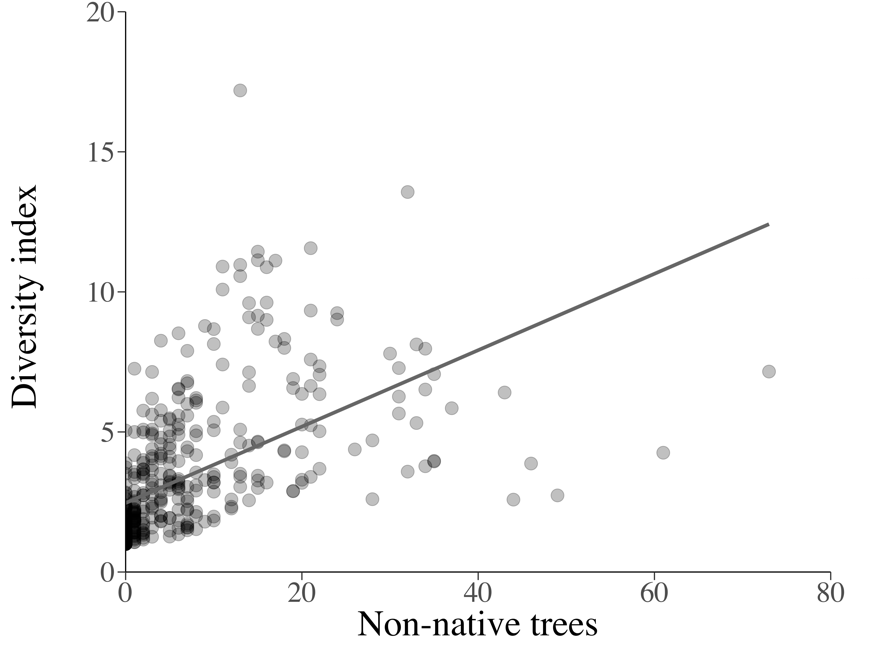


***Figure S6.* The relationship between the number of non-native trees and diversity index within territories.** While no co-linearity was found in the models (VIFs < 2), non-native tree species represent the majority of species present in the urban parks and thus relate to tree diversity (estimated as the inverse Simpson’s index).


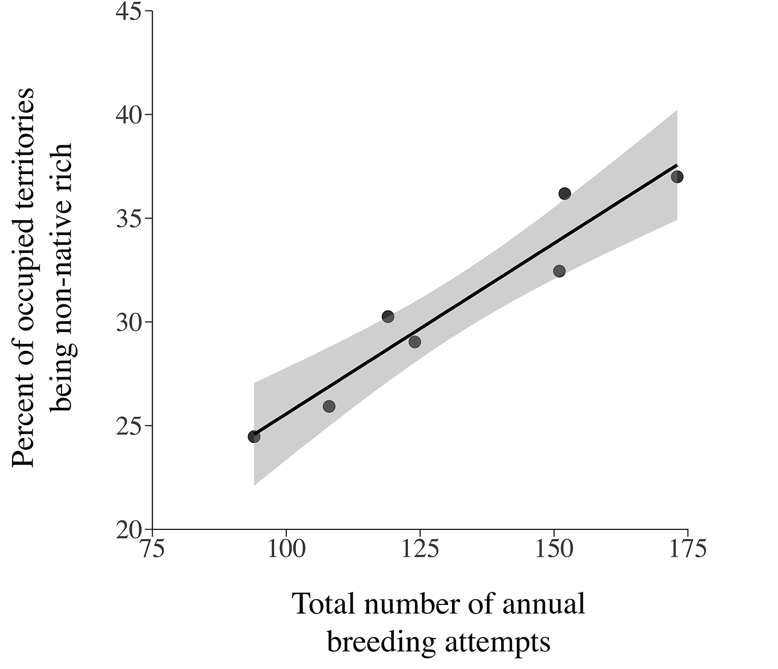


**Figure S7. Correlation between population size per year and non-native rich territories occupied.** To test if blue and great tits avoided occupying nest boxes situated in areas with high numbers of non-native trees in years when assumed competition between nest boxes were low, we tested the relationship between the percentage of territories found in areas with high numbers of non-native trees (y-axis) and total number of annual breeding events (x-axis). The linear model showed a significant correlation (F = 60.67, df = 1, p < 0.001) between the proportion of breeding attempts in territories with an above-average number of non-native trees and the total number of breeding attempts. A value of 50 % (y-axis) would indicate no preference between non-native rich and poor territories. Shaded area shows standard error of the mean (SE).
